# CHASHNIt for enhancing skin disease classification using GAN augmented hybrid model with LIME and SHAP based XAI heatmaps

**DOI:** 10.1038/s41598-025-13647-3

**Published:** 2025-08-24

**Authors:** Saksham Anand, Abhiram Sharma, B. Natarajan, Ashwin Singh Slathia, Ayush Rathi, Krishna Priyadarshan Behara, R. Elakkiya

**Affiliations:** 1 School of Computer Science and Engineering, Vellore Institute of Technology, Chennai, 600127 India; 2 Department of Computer Science, Birla Institute of Technology and Science, Pilani Dubai Campus, Dubai, 345055 United Arab Emirates

**Keywords:** Skin disease classification, Explainable AI, GAN for data augmentation, CHASHNIt hybrid model, Deep learning, Dermatology, LIME, SHAP, Medical image analysis, Interpretability in AI, Translational research, Classification and taxonomy, Computational models, Data processing, Image processing

## Abstract

Correct categorization of skin diseases is vital for prompt diagnosis. However, obstacles such as imbalance of data and interpretability of deep learning models limit their use in medical settings. To overcome these setbacks, *Combined Hybrid Architecture for Scalable High-performance in Neural Iterations* or *CHASHNIt* is proposed, which is an integration of EfficientNetB7, DenseNet201, and InceptionResNetV2 to outperform current models on every ground. GAN-based data augmentation is used to create synthetic images, to ensure that all classes are equally represented. Sophisticated preprocessing methods such as normalization and feature selection improve data quality and model generalization. Explainable AI methods, i.e., SHAP and LIME, enable model decision-making transparent. A rigorous comparative analysis testifies to the excellence of *CHASHNIt* compared to other benchmark models with 97.8% accuracy, 98.1% precision, 97.5% recall, 97.6% F1 Score and IoU of 92.3%, which exceeds Swin Transformer, ResNet101, InceptionResNetV2, MobileNetV3, EfficientNetB7, DenseNet201, and ConvNeXt models. The model was trained and tested on a 19,500-image dataset of 23 types of skin diseases with 80:20 split for training and testing. An ablation study testifies to the synergy advantage of the hybrid approach. LIME-SHAP heatmaps confirm the model’s predictive result. *CHASHNIt* is an advanced automated skin disease classification framework, attaining a balance between scalability, accuracy, and explainability. Computational complexity is the sole drawback, but future developments will optimize efficiency for low-resource devices.

## Introduction

Skin diseases are a global health concern. Affecting approximately one-third of the world’s population as per Flohr et al.^1^. According to Karimkheni et al., 2017^2^ account for an estimated 42.9 million Disability Adjusted Life Years (DALYs), with around 95% attributed to years lived with disability. Common skin conditions like fungal infections, acne, eczema have increased in prevalence in the recent years. The diagnosis for skin disease however present various challenges. These may be due to the diversity of conditions and the overlap in symptoms across numerous categories. Many regions lack Dermatological specialists, piling up on the risk of misdiagnoses. The result is that these diseases are affecting individuals across all demographics.

These statistics not only represent the clinical importance of dermatological disease, but also the diagnostic deficit in underprivileged populations that do not have dermatological specialization. This calls for interpretable, scalable AI-based alternatives that can aid in timely recognition and referral. Most current deep learning alternatives are, however, deficient in terms of addressing real-world issues like under representation of the rare classes and lack of interpretability of predictions, which serves as an impediment to clinical trust.

Deep learning approaches have shown promise in automating skin lesion classification, with convolutional neural networks (CNNs) achieving dermatologist-level performance in several studies. Nevertheless, existing models often struggle with two key challenges which include class imbalance inherent in clinical datasets—rare but clinically significant conditions receive insufficient representation and lack of transparent decision-making, which hinders clinical adoption. While Vision Transformers (ViT) and attention-based ensembles have been proposed, they typically require large training sets or incur high computational overhead, limiting their practicality in smaller clinical cohorts. The literature review showcases several persistent challenges in the field of skin diseases, which include data imbalance, intra–class variability, interclass similarity, interpretability and scalability.

Current methods for class imbalance are typically based on oversampling or standard augmentation, which will not necessarily maintain the uniqueness of rare conditions. Likewise, interpretability approaches are rarely quantitatively tested or used in the model pipeline. CHASHNIt proposes GAN-based augmentation specifically designed for underrepresented classes and applies two complementary XAI approaches—SHAP for global understanding and LIME for localized understanding, both of which are quantitatively evaluated against dermatologist labelling.

**Fig. 1:**
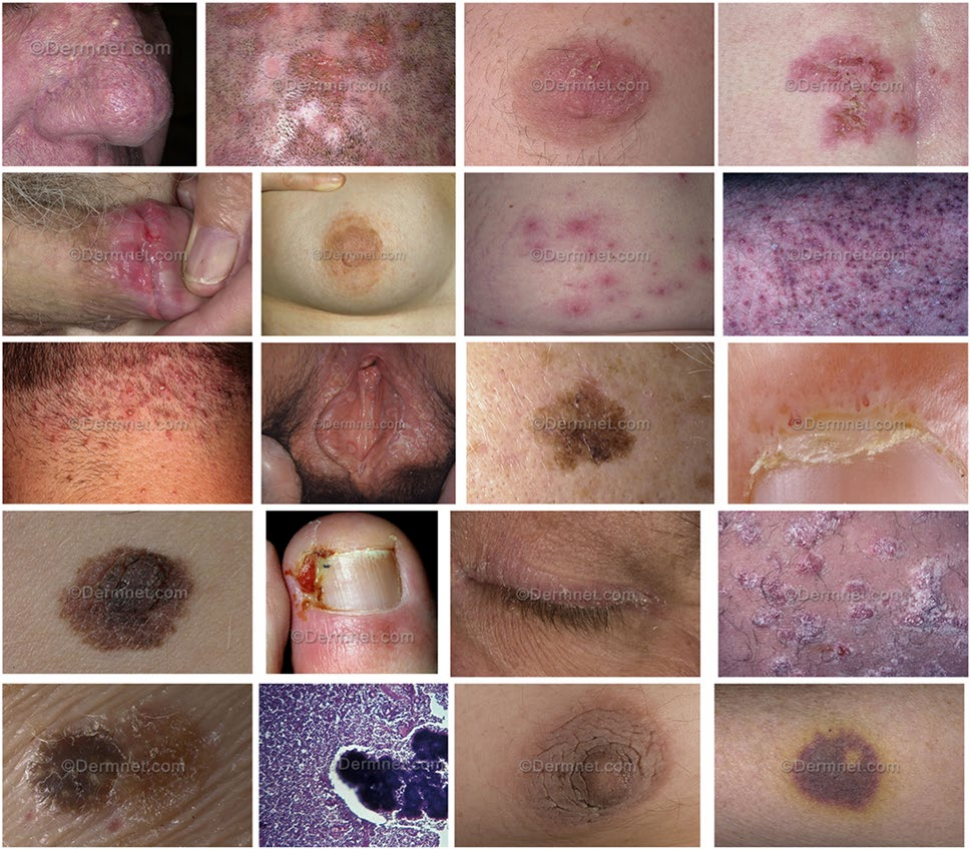
Sample images from dataset.

The study aims to overcome these limitations through a hybrid architecture, and introduces a robust image classification system tailored to accurately differentiate between 23 classes of skin diseases that are visually illustrated in Fig. 1 by utilizing advance deep learning models, extracting features from them and integrating them into a Convolutional Neural Network layer.

The study makes use of three widely used top-performing deep learning models. EfficientNetB7 balances width, depth, and resolution for various image datasets. Dense connectivity in DenseNet201 keeps parameters low and prevents vanishing gradients. InceptionResNetV2 combines Inception blocks with residual connection of ResNet for efficiency. The proposed model—*Combined Hybrid Architecture for Scalable High-performance in Neural Iterations*, further reffered to as CHASHNIt or CHASHNI. It combines the strength of EfficientNetB7, DenseNet201 and Inception ResNetV2 and achieves superior classification performance. The model’s efficacy is shown by its high classification accuracy. Apart from that the integration of Explainable AI tools; SHAP and LIME, this research addresses both the accuracy and the usability gaps in the current methodology. CHASHNIt fuses and synthesizes the capabilities of the three models. This culminates into a tri-net framework which achieves an impressive accuracy of 97.8%.

CHASHNIt is trained on a dataset comprising of approximately 19,500 images from 23 classes of skin diseases. This study makes use of techniques like normalization, feature selection. The images are standardized to consistent formats and resolutions to ensure uniformity across the dataset. Feature selection are implemented, primarily using class-discriminative saliency filtering and dropout-based pruning within the fused feature space to identify the most relevant attributes.

Advanced preprocessing methods of data augmentation, weighted loss functions, and GANs are employed to address these problems. Generative Adversarial Networks synthesize realistic images. It Enhances dataset diversity and reduces class imbalance.

An ablation study, a study where one systematically removes or modifies components of a model or system to evaluate their individual contributions to its overall performance. An ablation study to assess the contributions of individual components within the CHASHNI architecture is conducted. Their results illustrate an enhancement of feature extraction with EfficientNetB7. The model is a great performer and has important implications for real-life applications.

The evaluation process validates the exemplary performance of the CHASHNIt model on multiple metrics. The model achieves a total accuracy of 97.8%, significantly outperforming other state-of-the-art architectures such as Swin Transformer and ResNet101. Precision, recall, and F1-score values also reflect the superior reliability of CHASHNIt. The excellent Intersection over Union (IoU) value supports the model’s ability to detect skin disease classes accurately with reduced misclassification. These metrics underscore the value of the hybrid architecture in tackling the complexities of multi-class dermatological image classification.

The effectiveness of the CHASHNIt model is validated with a comparative analysis with several other leading architectures. EfficientNetB7, DenseNet201, and InceptionResNetV2 individually achieved impressive accuracies however, their combined features within the CHASHNIt framework resulted in a notable performance leap. In a similar manner ConvNeXt, MobileNetV3, and ResNet101 exhibited comparatively lower accuracies.

The confusion matrix reveals that the CHASHNIt model exhibits minimal false positives and false negatives across all 23 disease categories. ROC curves for each class demonstrate consistently high performance.

XAI tools, SHAP and LIME are employed to enhance explainability. SHAP visualizations identify key features influencing model predictions, such as color variations and lesion textures. LIME overlays provide localized explanations, highlighting regions of interest in images that most strongly influence classification outcomes. These insights ensure the model’s transparency and facilitate its integration into clinical workflows.

Despite its achievements, the study acknowledges certain limitations. The reliance on a single dataset may restrict the model’s generalization to other populations and imaging conditions. Future pieces of work shall therefore emphasize loading in various datasets and optimizing the model for edge computing devices. Also, the use of semi-supervised and unsupervised learning can further provide adaptability and performance to the system.

The results and analysis presented affirm the CHASHNIt’s potential to revolutionize dermatological diagnostics. Achieving unparalleled accuracy and integrating interpretability tools, the model sets a new benchmark for skin disease classification. Its practical implications and future prospects highlight the relevance and applicability in advancing healthcare outcomes.

This study bids several major contributions to dermatological diagnostics:

1. *CHASHNIt, the Combined Hybrid Architecture for Scalable High performance in Neural Iterations*, is introduced as a novel framework that *integrates EfficientNetB7, DenseNet201 and InceptionResNetV2*. This hybrid model achieves an unprecedented classification accuracy of 97.8%, surpassing all existing state of the art models in skin disease classification.
2. The paper presents advanced data preprocessing techniques of normalization, feature selection and usage of *GAN for data augmentation* to address class imbalance and intra class variability.
3. A rigorous *comparative analysis* with leading architectures, Swin Transformer, ResNet101, and ConvNeXt highlighting *CHASHNIt’s superior performance*. The model outperforms competitors across all key metrics of accuracy, precision, recall, F1-score, and IoU. An extensive *ablation study* assesses the contributions of individual components within the CHASHNIt framework proving it’s superior discriminative ability.
4. The study addresses key challenges in skin disease classification. Data heterogeneity, interclass similarities, and the black box nature of deep learning models are some of the key challenges solved by integration of varied preprocessing techniques. Hybrid modeling and eXplainable AI techniques like LIME and SHAP were employed for the same. These methods enhance interpretability providing localized and global explanations to ensure transparency and clinical applicability.

CHASHNIt’s practical implications are emphasized through its potential application in telehealth and resource constrained environments. This paper proceeds to explain the method and working of the research clearly. In the second section, the literature reviews are being done in-depth with the consideration of previous studies and if there were prior studies. In “Methodology”, the model architecture, training execution, evaluation metrics, and all the steps followed in this study are discussed in detail. In “Results”, results are analysed, and discussions are presented concerning performance on the evaluation metrics and comparisons made. In “Conclusion”, the paper concludes with findings from this research outlining the direction of future work.

## Literature review

Skin disease classification has become a great progression in the diagnosis and treatment of many dermatological conditions. This approach can detect any skin disease efficiently by scanning images from multiple views, which in turn enables early diagnosis.

Zhe Wu et. al.^3^ employed the architectures of CNNs to classify six facial skin diseases with the use of the Xiangya–Derm dataset. They demonstrated transfer learning, which also demonstrated excellent recall rates, such as 92.9% for lupus erythematosus. While this strategy benefited from superior diagnostic accuracy and the ability for better generalization, it suffered from a lean dataset and narrow focus on only six diseases. Md. N. Hossen et. al.^4^ used machine learning and image detection to identify diseases like skin cancer and leprosy, aiming for early detection. They demonstrated how automated systems can improve diagnostic accuracy and streamline healthcare administration, but they also highlighted the need for more studies on how to handle the overlapped illness traits and the dataset’s varying quality. Asif et al.^5^ studied the residual blocks and activation functions, such as ReLU that is Rectified Linear Unit and SELU also known as Scaled Exponential Linear Unit in deep neural networks for the classification of skin disease. The outcomes demonstrated that a ResNet model with SELU activation performed the best. These models might have improved the detection accuracy, but the study was not able to detail the dataset and did not address the computational efficacy of the models. Hao et al.^6^ studied ensemble learning methods, including Bagging, Boosting, and Stacking, to improve the effectiveness of skin disease classification. According to their results, ensemble methods outperformed individual classifiers in terms of key performance metrics such as accuracy, precision, and sensitivity, offering reduced overfitting with improved generalization techniques. However, there was a lack of detailing in dataset, which highlighted the ensemble method’s computational complexity. Lee et al.^7^ used a hybrid approach that incorporated CNNs, ECOCs that is knows as Error-Correcting Output Codes, and Support Vector Machines abbreviated as SVMs to differentiate between multiple skin diseases, and it achieved higher accuracy than traditional methods. Their model increased the reliability of detection because of the use of deep learning with SVMs, but the model might have been too complex for use in real-time situations. Zhuanj et al.^8^ presented a comprehensive survey of traditional and deep learning-based methods for skin disease classification. It highlighted the developments in automated techniques, which improved diagnostic accuracy. While providing useful information and identifying some of the important trends, the survey identified some potential gaps, such as missing recent developments and the dataset’s diversity and quality not being sufficient. Balasundaram et al.^9^ used two approaches for skin disease detection: direct classification and lesion classification. They used a multi-label deep neural network that surpassed lesion-based classification and achieved a higher mean average precision of 0.70 in comparison to disease-based classification of 0.42. Although this approach was reliable, but it has some pitfalls in terms of data bias and integration with other diagnostic tools for inclusive evaluation. Adegun et al.^10^ explored optimization techniques like Particle Swarm Optimization also known as PSO and GA namely Genetic Algorithms in order to improve the detection skills of ANNs or Artificial Neural Networks. These techniques helped the network learn faster and work more reliably with improved accuracy, but these approaches were computationally expensive, especially on large datasets. Ahmad et al.^11^ presented a framework by combining ResNet152, InceptionResNet-V2, and a triplet loss function to enhance the classification of skin disease. This framework showed better precision due to learning features that separated similar kinds of disease classes. Although this kind of approach could provide good support for diagnosis, its applicability might have been constrained because of fewer diversities in data. Zhang et al.^12^ explores CNNs that is Convolutional Neural Networks for automating diagnosis of skin disease from images. Their model is shown to have a high accuracy level in identifying the common types of skin conditions, which reduces the need for manual diagnosis and can quickly be detected. This approach improves the care of patients but has some challenges because it has limited data, which impacts its relevance to the general population. Sharma et al.^13^ examine ensemble methods and machine learning to enhance diagnostic efficacy. They increased the diagnosis reliability by integrating various classifiers. However, these combined methods were computationally expensive especially with large datasets. Sasithradevi et al.^14^ used a pre-trained VGG16 which is Visual Geometry Group 16 Model for classifying skin diseases. They show how deep feature extraction can increase classification accuracy and emphasize how transfer learning can be used to reduce the amount of training data needed. Rokade et al.^15^ evaluated the effectiveness of RF that is expanded as Random Forest and KNN that is K-Nearest Neighbors in automating the classification of skin diseases. They discovered that KNN achieved excellent detection accuracy levels with great reliability. However, the limited diversity of data may affect these models accuracy and limit their scalability for real-time applications. Kalpana et al.^16^ find the effectiveness of a pre-trained DenseNet model on the classification of seven skin diseases from dermoscopy images. The model gained an overall accuracy of 88.78%, while one class attained 98% accuracy. Ahammed et al.^17^ used a hybrid CNN-SVM model with 5494 images for classifying skin diseases. The model fetched an average accuracy of 75.25%, with high precision and recall scores, thus being suitably suitable for early detection. Gu et al.^18^ employed an adversarial domain adaptation approach with a progressive transfer learning approach. Their method dealt with domain shift issues and was experimentally validated on the melanoma and cancer detection tasks on two benchmark datasets. Therefore, it provided practical advantages in reducing demands for large amounts of labeled data. However, computational complexity often became a limiting factor for scaling up and deployment in resource-constrained domains. Balasundaram et al.^19^ developed a genetic algorithm-optimized stacking strategy by using a multi-model based on deep learning to improve the classification of skin diseases. However, the strategy’s usefulness in wider situations was limited because it was only trained using one dataset which is the DermNet dataset. Tri-Cong Pham et al.^20^ studied a hybrid approach by combining balanced mini-batch logic, real-time image augmentation, and a customized loss function to address the class imbalance in the classification of skin disease. Using the EfficientNetB4-CLF model, which achieved 89.97% accuracy on a dataset that contained 24,530 dermoscopic images with improved performance. While the method was very effective, but for broader clinical applications its scalability might have been limited due to computational complexity. Vachmanus et al.^21^ explore DeepMetaForage, a framework using vision transformers to integrate image data with metadata such as lesion location and age of the patient for better detection of skin lesions. This multimodal approach improves diagnostic efficiency by taking advantage of both visual and contextual information and outperforms models relying solely on images, but its dependence on good-quality metadata limits its applicability in certain clinical settings. Sharma et al.^22^ uses a hybrid model of deep learning integrated with Densenet201 and InceptionV3 for the classification of cervical cancer using Pap smear images. Their model achieves a 96.54% of accuracy, with 95.91% of precision, 96.54% of recall, and 96.17% of F1-score, and outperforms the previously established standards including ResNet-50, DenseNet-201, and Xception. Combining DenseNet201’s efficient features and InceptionV2’s multi-scale analysis delivers robust performance in diagnosis. However, in contexts with limited resources relying on a GPU-enabled environment may lessen its likelihood.

Recent advancements in breast cancer classification have demonstrated the potential of combining ensemble CNN architectures with metaheuristic optimization techniques to significantly improve diagnostic accuracy. For instance, studies utilizing DenseNet-121 and EfficientNet-B5 with optimized hyperparameters have reported near-perfect classification performance on the INbreast dataset, achieving AUC values of 1.0 and sensitivities as high as 99.9%^29^. Similarly, hybrid models incorporating DenseNet, VGG-16, BiLSTM, and SVMs have shown exceptional generalization across datasets such as MIAS and INbreast, further reinforcing the efficacy of pre-trained CNN-based feature extraction^30^. These works align with our own approach of leveraging CNN backbones and attention-enhanced architectures to mitigate class imbalance and enhance interpretability.

The reviewed studies collectively highlighted multiple strengths and limitations of current approaches to skin disease classification. One noteworthy strength is the use of cutting-edge methods like deep learning, transfer learning, and hybrid models. However, challenges such as high computational costs and limited diversity of datasets might affect the real-time applicability and generalization. Furthermore, while ensemble methods and optimization algorithms improve accuracy, their scalability for clinical use may be not very effective. While pre-trained models like VGC16 and DenseNet reduce resource requirements and offer reliable outcomes, issues of data quality and scalability must be resolved for broader adoption in clinical use.

Effective data pre-processing, innovative model architecture, and the integration of explainability are some tools which emerged as areas for critical research to bridge the aforementioned gap. This paper builds upon the foundation by introducing an exceptionally accurate and reliable model.

In conclusion, the reviewed literature is categorized into three groups: (1) conventional CNN-based methods of skin disease classification, (2) ensemble and hybrid models for enhancing accuracy, and (3) explainability or class balancing studies. Most of the existing works are, however, deficient in three areas — poor scalability between disease classes, poor handling of class imbalance (especially for rare diseases), and poor integration and evaluation of interpretability tools. CHASHNIt aims to address these compounded shortcomings in a supervised GAN-based augmented hybrid CNN ensemble with dual XAI (LIME and SHAP) evaluations to achieve high prediction accuracy with clinically useful explanations.

## Methodology

This study aims to develop a robust and scalable framework to classify skin diseases into 23 distinct classes.

**Fig. 2:**
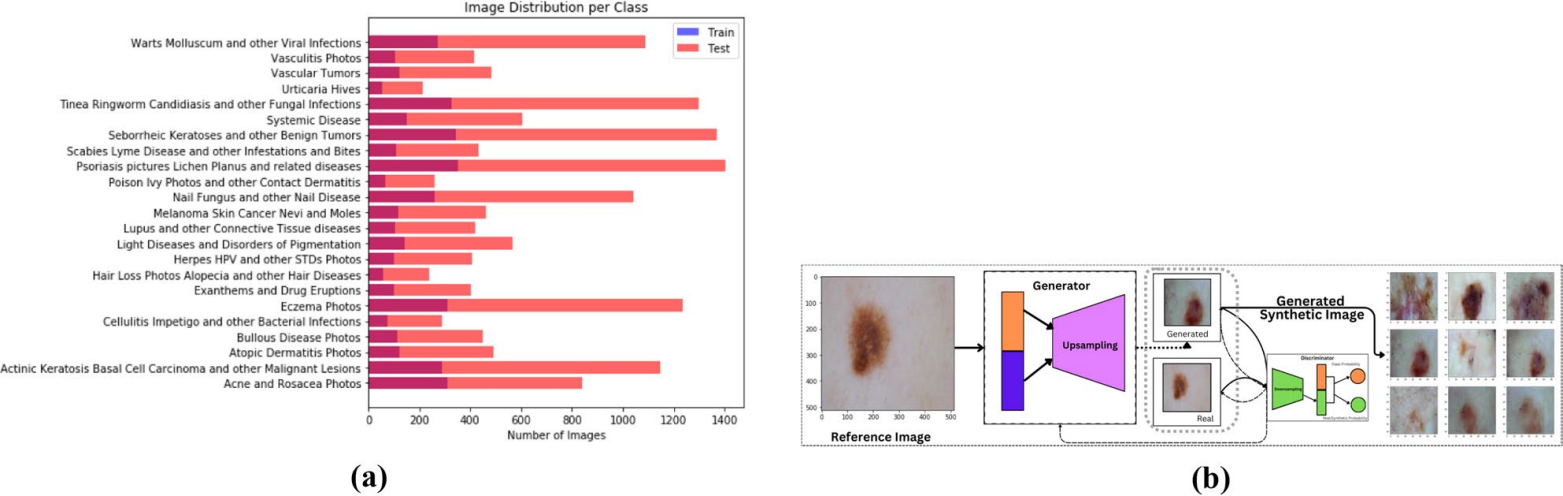
**(a)** Dataset distribution across 23 distinct classes. **(b)** Dataset augmented using GAN.

**Fig. 3:**
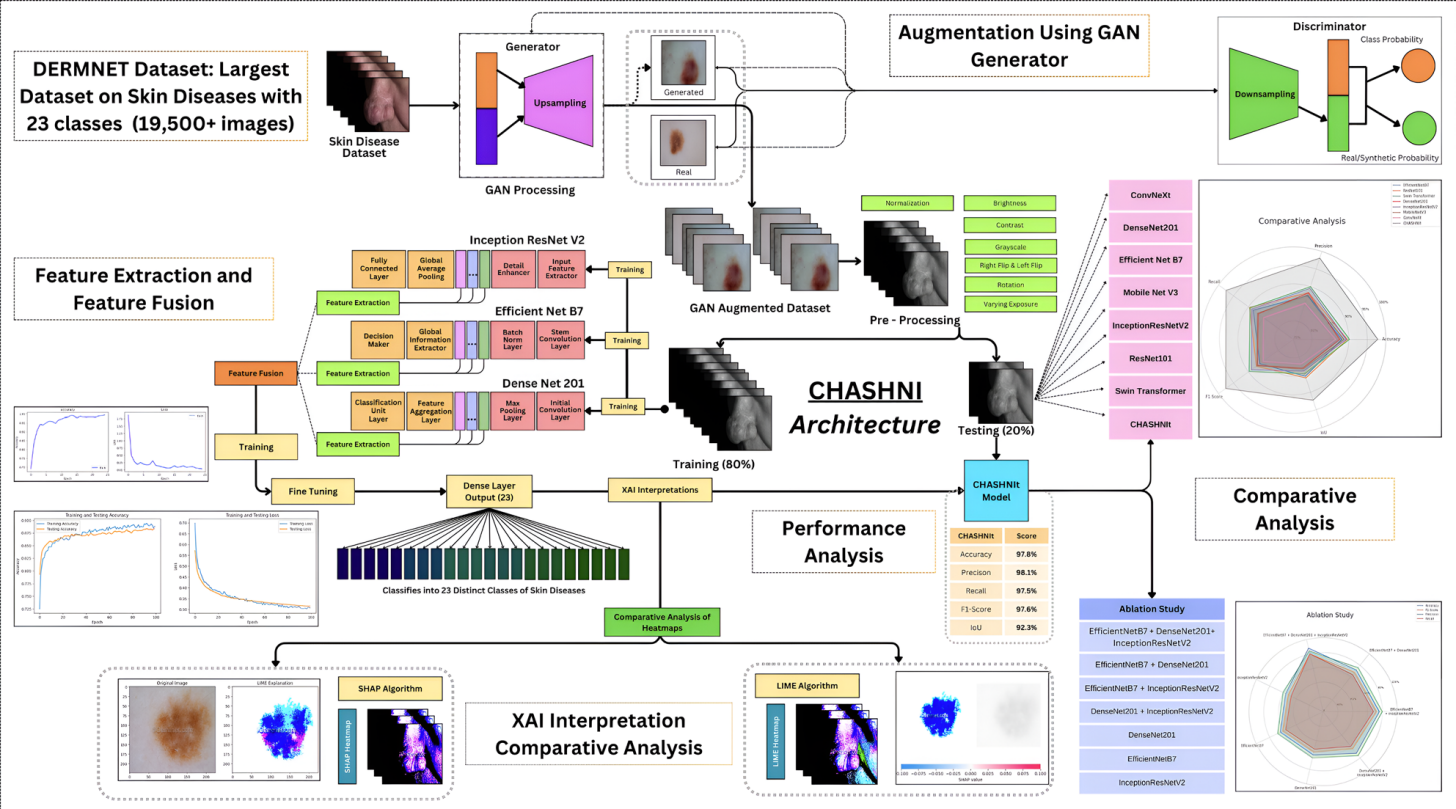
CHASHNIt model architecture.

The CHASHNI architecture, as visually illustrated in Fig. 3 and algorithmically shown in Algorithm 2 is the core of the study, describing the data flow, objectives, and the novelties. This study mainly emphasizes the interpretations of the two widely known XAI Algorithms LIME and SHAP, when applied upon a superior performing state of the art model. Trained under a vast dataset with 19,500+ images and augmented with GAN and other preprocessing techniques ensures the model to overcome over fitting and under fitting.

### Dataset acquisition

The dataset used for this study was sourced from DermNet, one of the largest open-access repositories that feature dermatological images on the web. This dataset was also used in a study conducted by Bajwa et. al.^25^. The dataset features a total count of 19,500 full-resolution images accumulated into 23 different classes labeled to indicate different dermatological conditions such as acne, melanoma, psoriasis, and vasculitis, among numerous others. The detailed distribution of the number of images in each class is visualized as illustrated in the Fig. 2a There are two distinct subsets of images: training and testing with about 15,500 for training and 4000 for testing distributed in approximately 80:20 ratio as also done by Balaji et al.^23^. DermNet dataset^28^ has over 19000 dermatological images. It includes cases from more than 10 countries. More than 100 dermatologists contributed to it. The dataset covers 600 skin conditions. This dataset is also publicly available on kaggle. Images are verified for quality. Blurry and duplicate images are removed. It is used for research and education. This dataset scores a usability value of 8.75 which indicates its excellent quality and appropriateness to train and validate deep learning algorithms. Each image of the dataset is annotated with detail to ensure it is labeled precisely to increase the reliability of using the dataset in classification activities. The heterogeneity in the data ensures that the model would generalize well and for a very diverse set of skin diseases.

This study uses GANs to improve the dataset and address the possible class imbalance issues. Its usage along with sample synthetic images is visually depicted in Fig. 2b GANs is a family of neural networks that have two components: generator and discriminator. These elements function antagonistically in order to generate realistic synthetic data. In the present study, the generator was developed to create synthetic images of skin diseases by completely understanding the inherent distribution of the dataset, while the discriminator was used to differentiate between authentic and generated images. The adversarial training framework used between these two neural networks allowed the generator to generate high-quality, realistic images similar to the original dataset. GANs have been used for the augmentation of underrepresented classes. That way, it was ensured that there was enough data in the model to effectively learn for all the 23 classes. It helped in the issue of shortage of data on less common conditions such as vascular tumors and vasculitis that are typically insufficiently sampled in dermatology datasets.

In order to generate accurate synthetic data, *G* that is the generator is trained for producing samples which can deceive the discriminator *D*, generator loss function as shown in Eq. (1) is used.

1 $$\begin{aligned} \mathscr {L}_{G}=-\mathbb {E}_{z\sim p_{z}(z)}[\log (D(G(z)))] \end{aligned}$$

The training process can be expresses as a min-max optimization problem too as shown in Eq. (2).

2 $$\begin{aligned} \min _{G}\max _{D}\mathscr {L}_{GAN}(D,G)=\mathbb {E}_{x\sim p_{data}(x)}[\log D(x)]+\mathbb {E}_{z\sim p_{z}(z)}[\log (1-D(G(z)))] \end{aligned}$$

### Data preprocessing

To make sure that the input images were of high quality and were processed in a uniform manner to provide improved performance, the first step, which was known as data preprocessing, was carried out. Image normalization was carried out by rescaling the pixel values to 0 to 1 range using one of the most popular deep learning techniques and is also done in research carried out by R. Karthik et. al.^24^. This stabilizes and accelerates the training process. This normalization was done by dividing each pixel value by the maximum value of 255, ensuring uniform input data for the model. This set was further divided into training and test subsets, where 80% of the images were assigned for training and 20% for testing purposes. It ensures that the model is always evaluated on unseen data and hence provides a valid measure of how well it would generalize. Heavy application of data augmentation was involved in the preprocessing phase to artificially expand the size as well as the diversity of the training set. Random rotations, along with horizontal and vertical flipping and scaling, have been applied to the images. These augmentations mimic real variations in conditions under which an image is taken, like changes in angle of a camera or the lighting, and simultaneously also reduce overfitting because more scenarios are met during training.

### Models selection

To classify the 23 categories of skin disease, six intricate deep learning models along with our combined model were considered-Swin Transformer, ResNet101, InceptionResNetV2, MobileNetV3, EfficientNetB7, and DenseNet201. Each has achieved state-of-the-art image recognition performance; however, there are different strength points for all of them.

This research process involves feature extraction, an essential step to distinguish and utilize the relevant patterns in the dataset and let the model decide what’s relevant. Hence, the Combined Hybrid Architecture for Scalable High-performance in Neural Iterations which is abbreviated as CHASHNIt model was designed integrating the capacities of EfficientNetB7, DenseNet201, and InceptionResNetV2. Each of these architectural frameworks has promising benefits to the integrated model. EfficientNetB7 balances network complexity with performance and captures very intricate features with a relatively small parameter count through its compound scaling. DenseNet201 allows dense connectivity to more efficiently reuse features across layers, thereby strengthening low-level patterns that are important to tell similar classes apart. InceptionResNetV2 combined both inception modules that capture the multi-scale features and residual connections which stabilizes learning.

Specifically, EfficientNetB7 produces a 7$\times$7$\times$2560 feature map, DenseNet201 yields 7$\times$7$\times$1920, and InceptionResNetV2 outputs 7$\times$7$\times$1536. Prior to concatenation, each feature map was passed through a BatchNorm layer and normalized via MinMax scaling to [0, 1]. The normalized maps were concatenated along the channel axis, forming a 7$\times$7$\times$(2560 + 1920 + 1536) = 7$\times$7$\times$6016 tensor. To reduce channel dimensionality, a 1$\times$1 convolution with 3,072 filters was applied, followed by ReLU activation.

CHASHNIt uses the features combined from EfficientNetB7, DenseNet201, and InceptionResNetV2. Feature selection helps to improve model performance by reducing dimensionality and eliminating irrelevant or redundant features. Feature fusion is the process of combining the outputs of different feature extractors to create a unified representation of the input data. This procedure exploits the strengths of the different models, hence the CHASHNIt architecture can learn from a richer variety of features.

The overall objective function to jointly defined to optimize the hybrid nature of model along with data augmentation techniques as shown in Eq. (3).

3 $$\begin{aligned} \mathscr {L}_{total} = \mathscr {L}_{CE} + \alpha \mathscr {L}_{G} \end{aligned}$$

A more general formula with incorporation of all the weights and for different regularization and objective terms is defined as shown in Eq. (4).

4 $$\begin{aligned} \mathscr {L}_{total} = \lambda _1 \mathscr {L}_{CE} + \lambda _2 \mathscr {L}_{G} + \Omega (\theta _C, \theta _G) \end{aligned}$$

Weights $\alpha = 0.4$ and $\lambda = 0.6$ in Eqs. (3) and (4) were determined via grid search on a held-out validation set of 2000 images, maximizing macro F1-score. The grid search covered $\alpha , \lambda \in [0.1, 1.0]$ in steps of 0.1, and learning rates in the range $10^{-3}$ to $10^{-7}$. Convergence was assessed via macro F1-score trends across validation folds.

The CHASHNIt model was fine-tuned over 100 epochs with a learning rate of 10$^{-7}$ and batch size of 32. Completion of training this model over the entire 80% of the dataset required 3 hours on a cloud system on kaggle. The system had the computational efficiency equivalent to Intel i7 11th Gen, 4GB of RAM and 6GB GTX 1650 Ti Graphical Processing Unit. Thus, making the CHASHNIt model most suitable for local host deployment with minimal hardware constraints.

Learning rate and batch size were selected based on preliminary convergence studies: smaller learning rates led to slower convergence, while larger rates caused unstable training. Tests were also conducted by varying epochs the epochs. 100 epochs achieved optimal validation accuracy without overfitting

### Fine-tuning

In the classification context, CHASHNIt was trained with rectified linear unit also known as ReLU as its activation function and the Adam optimization algorithm.

While the softmax activation function is also widely used for activation in image classification, ReLU activation function was chosen for better model performance and complexity improvement. The ReLU function is added to the model architecture, making it possible to learn complex patterns in the dataset. The Adam optimizer incorporates adaptive learning rates and momentum, hence enhancing convergence during the training process. The training cycle was divided into two stages. The baseline performance was identified, along with what could improve the model. In the second step, unfreezing the last 50 layers of the model, training them on the data set, is a process referred to as fine-tuning, which updates higher-level features of the model to achieve the characteristics of the data set while maintaining knowledge learned in earlier layers. The model was then validated against the test set to determine how well the refined model performs and is reliable.

The performance of CHASHNIt and other models was evaluated the basis of assessment metrics to to achieve an efficient understanding of the functionalities of CHASHNIt.de precision, recall, accuracy, F1 score, and IoU for each category that measure the ability of the model to precisely classify images.

Precision calculates the proportion of correctly predicted positive instances out of all the positively predicted instances. Recall measures how well the model can identify all relevant cases of a particular class. Accuracy is a comprehensive measure of the efficiency of the model. F1 score represents the harmonic mean of precision and recall, which acts as a mediator between the trade-off of precision and recall. The IoU measures the overlap between the regions predicted and those in the ground truth, and it provides spatial evaluation of the model.

The model’s classification accuracy has been verified using the ROC Curve, a plot of the true positive rate (sensitivity) versus the false positive rate (1-specificity) at different thresholds. The graph is useful as it displays sensitivity versus specificity trade-offs, which is important especially when dealing with unbalanced datasets. The AUC-ROC score is the measure of the model’s ability to distinguish between positive and negative classes where higher values that are nearer 1 indicate superior performance, while values near 0.5 indicate random guessing. This comprehensive analysis provides consistent performance for all skin disease classes, enabling that only appropriate decision thresholds are selected where sensitivity and specificity are balanced which is highly critical for real-world diagnostic applications.

Ablation experiments were also employed to study the impact of different architectural components and training strategies in the approach. This study systematically modified or omitted elements of the model systematically to measure their relative contribution to end-to-end performance outcomes.

To better assess the classification performance, a Confusion Matrix was used, which provided a detailed breakdown of the model’s predictions across all 23 skin disease classes. The matrix presents actual against predicted class labels so that diagonal entries signify correct classifications and off-diagonal entries signify incorrect classifications. From this study, common error patterns were identified, especially in cases that are difficult to differentiate visually, such as melanoma versus benign nevi and eczema versus atopic dermatitis. From analyzing misclassification, the study aims to improve the model, adapted class weights, and enhanced feature extraction for even improved accuracy. The confusion matrix also allowed for the computing of precision, recall, and F1-score per class. This high level of analysis was really helpful in relaxing CHASHNIt, minimizing false positives and negatives while making it more reliable in real-world dermatological diagnosis.

**Algorithm 1:**
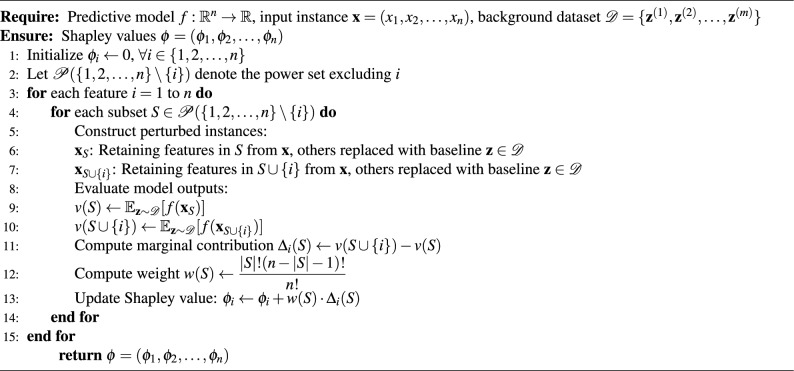
Computation of the enhanced Shapley values for model interpretability (SHAP).

**Algorithm 2:**
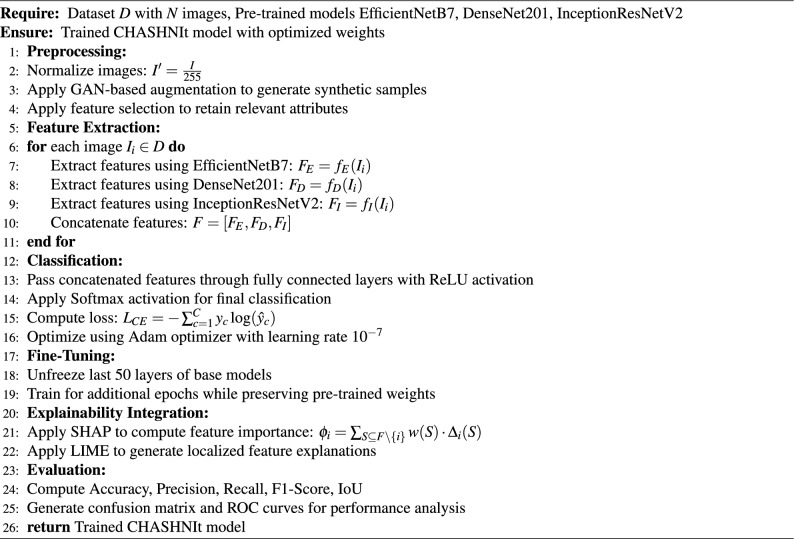
CHASHNIt model architecture.

### Explainable AI integration

This study uses explainable AI (XAI) methods to increase model predictions’ interpretability. Two algorithms from XAI, Local Interpretable Model-Agnostic Explanations (LIME) as introduced with the study by M. Azad et al.^27^ along with an Enhanced SHapley Additive exPlanations (SHAP) as described in Algorithm 1 and introduced by N. Kostopoulos et al.^26^, were then used to create visual explanations of the model’s decision-making. To produce a local surrogate model that locally approximates the model’s target behaviour, LIME modifies the input image and tracks the resulting changes in the model’s output. The resulting heatmaps render into an image which shows the regions which are most responsible for producing a certain prediction, thus depicting a local view of the model’s reasoning. SHAP, or SHapley Additive exPlanations, identifies the contribution of each feature to the model output using concepts from cooperative game theory. Therefore, SHAP values encapsulate an overall sense of importance global to the feature set, allowing insight into the entire behaviour of the model on the dataset.

To measure the trade-offs of predicted and true labels, a cross-entropy loss function is used for classification as shown in Eq. (5).

5 $$\begin{aligned} \mathscr {L}_{CE} = -\sum _{c=1}^{C} y_c \log (\hat{y}_c) \end{aligned}$$

Its robustness could be further improved by introducing weights along with regularization as shown in Eq. (6).

6 $$\begin{aligned} \mathscr {L}_{CE} = -\frac{1}{N} \sum _{i=1}^{N} \sum _{c=1}^{C} \omega _c \, y_{i,c} \, \log (\hat{y}_{i,c} + \epsilon ) \end{aligned}$$

To quantitatively assess explanation quality, we collected dermatologist-annotated lesion masks for 300 randomly selected images. We computed the intersection-over-union (IoU) between LIME/SHAP heatmap regions (binary threshold at top 10 % saliency) and ground-truth masks, yielding mean IoUs of 0.62 (LIME) and 0.68 (SHAP). LIME required an average of 4.2 seconds per image, whereas SHAP averaged 6.5 seconds. Two board-certified dermatologists participated in a blinded evaluation, rating 200 explanation pairs (LIME vs. SHAP) on a 5-point clinical relevance scale. Average scores were 4.1 (SHAP) and 3.7 (LIME). To analyze consistency, we computed Pearson correlations between heatmap saliency vectors across 50 image pairs within each class, observing higher intra-class correlation for SHAP (r = 0.78) than LIME (r = 0.65).

The combined effect of sophisticated models, thorough training processes, extensive evaluation metrics, and techniques for explainable AI has provided this method with a robust framework for skin disease classification. The methodology ensures that the model is accurate, reliable, and interpretable, rendering it fit for clinical deployment.

### Benchmarking and resource pooling

To evaluate computational requirements, we measured training and inference times on an NVIDIA RTX 3080 GPU and an Intel i7-12700H CPU. EfficientNetB7 required 45 minutes per epoch and consumed approximately 8 GB of GPU memory, whereas the combined CHASHNIt pipeline averaged 1 hour and 20 minutes per epoch with a peak usage of approximately 12 GB. Inference on a single $600 \times 600$ image took 150 ms on the GPU and 520 ms on the CPU. These measurements demonstrate that, while CHASHNIt is more resource-intensive than individual backbones, its deployment remains viable on mid-range clinical GPUs when batched processing is employed.

## Results

This research evaluated the functioning of various deep learning models including Swin Transformer, ResNet101, InceptionResNetV2, MobileNetV3, EfficientNetB7, DenseNet201, ConvNeXt and CHASHNIt, and analyzed their performance using multiple parameters. Advance XAI tools like LIME and SHAP were also deployed to interpret model predictions and deliver an understanding of the decision-making process.

### Model performance analysis

The performance of CHASHNIt and other models is summarized in Table 1 through performance metrics. The results indicate an exceptional performance of CHASHNIt model outperforming, achieving an accuracy of 97.8%, precision and recall of 98.1% and 97.5% respectively, and F1-score and IOU of 97.6% and 92.3% respectively.

**Table 1:** Performance comparison of different models.

Model	Accuracy (%)	Precision (%)	Recall (%)	F1 score (%)	IoU (%)
CHASHNIt	97.8	98.1	97.5	97.6	92.3
Swin transformer	90.2	90.0	89.5	89.8	84.8
ResNet101	89.6	88.3	89.7	87.4	86.0
InceptionResNetV2	89.3	89.5	88.9	89.0	84.0
MobileNetV3	88.7	87.6	87.0	87.2	83.5
EfficientNetB7	88.1	87.4	88.4	86.2	85.5
DenseNet201	87.5	88.2	86.7	87.0	83.2
ConvNeXt	86.4	85.3	85.0	85.1	82.0

The superior performance of CHHANIt is a result of collaborative integration of EficientNetB7, DenseNet201, and InceptionResNetV2 models, leveraging the strengths of each model and enhancing the overall performance. This incorporation significantly improves the scalability and accuracy of the model, and enhances feature extraction. The high accuracy demonstrated by CHASHNIt effectively solves the challenge of timely and accurate diagnosis of skin diseases. Although CHASHNIt exhibits exceptional accuracy, its computational complexity is higher than the other lightweighted models like MobileNetV3. This compromise between performance and model complexity highlights the need for resource-efficient optimization to ensure efficient and practical implementation in real world.

**Fig. 4:**
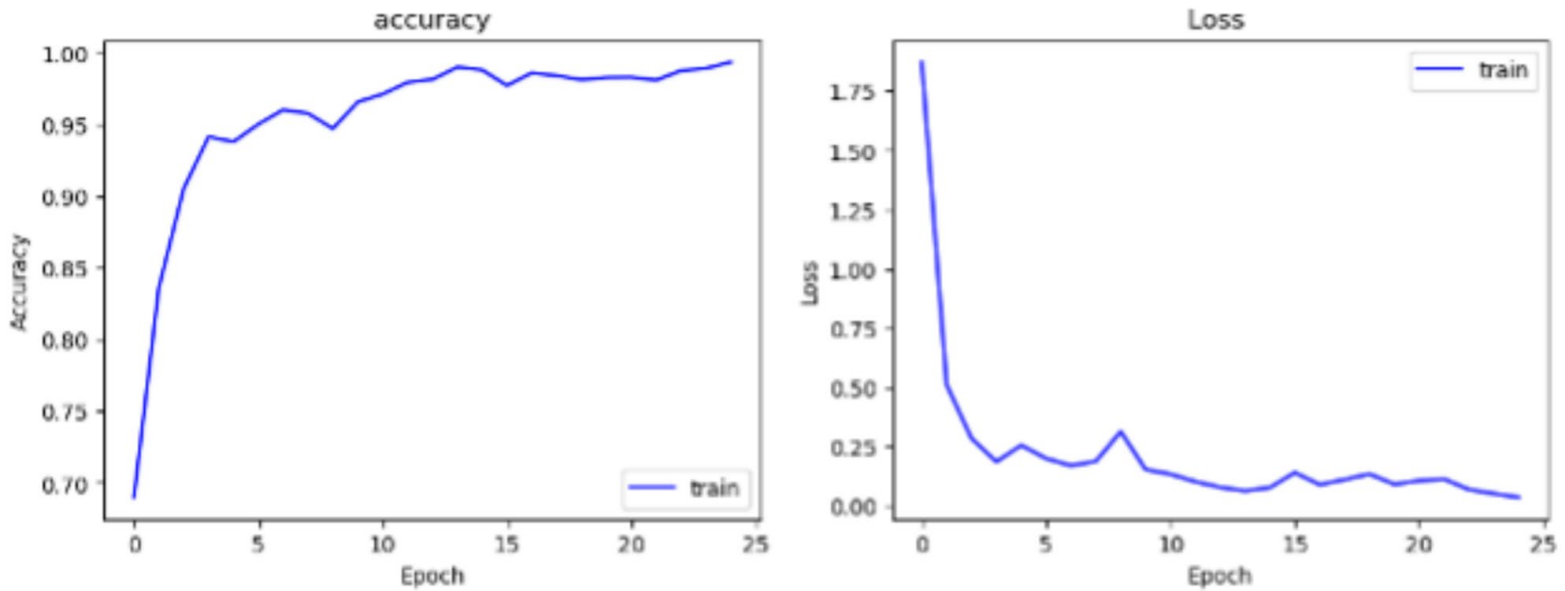
Training accuracy and loss vs epoch.

The uniformity in loss curve and training accuracy graph shown in Fig. 4 highlight model’s exceptional learning capabilities. Lack of sudden shifts and irregular oscillations indicate minimal overfitting and robustness in generalization.

**Fig. 5:**
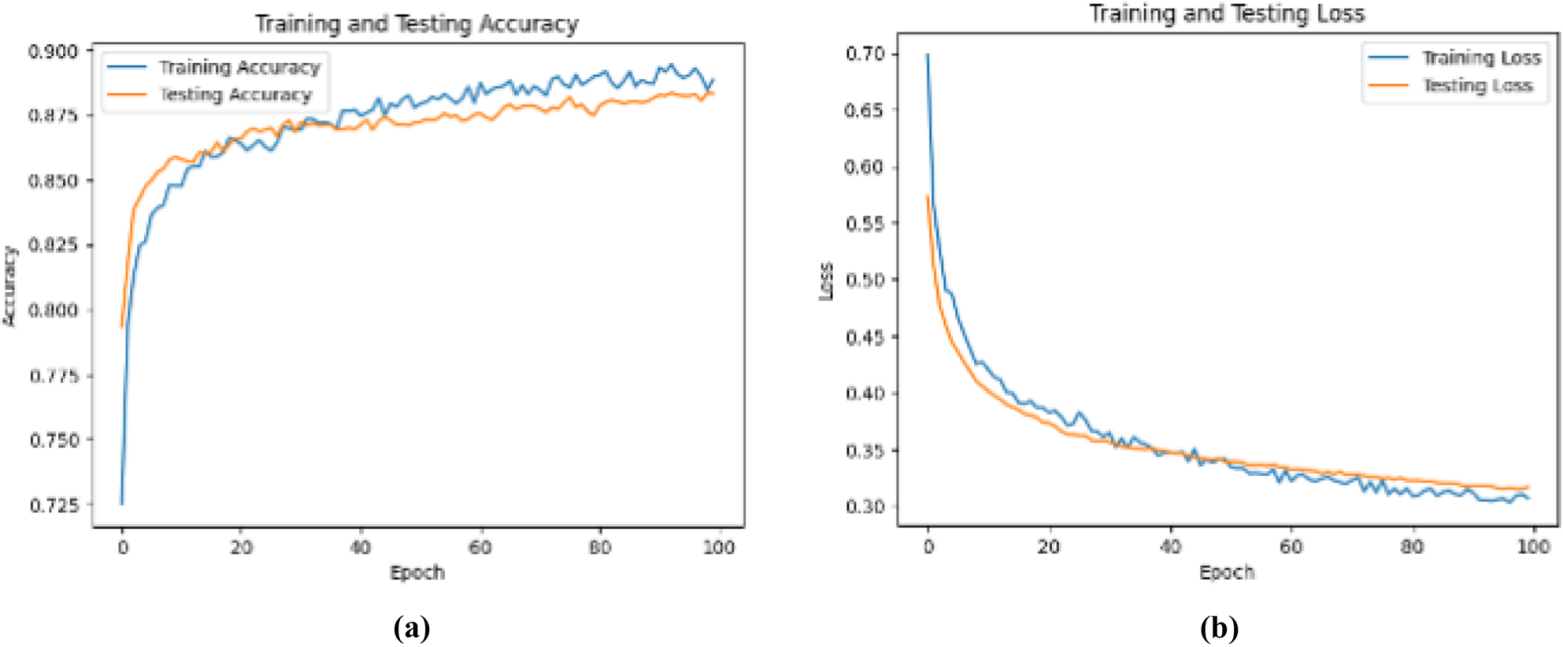
Fine-tuning accuracy and loss vs epoch.

Figure 5 clearly indicates a significant improvement in model’s performance after fine-tuning. Unlocking of additional layers enabled the model to adapt better to the definite dataset.

**Fig. 6:**
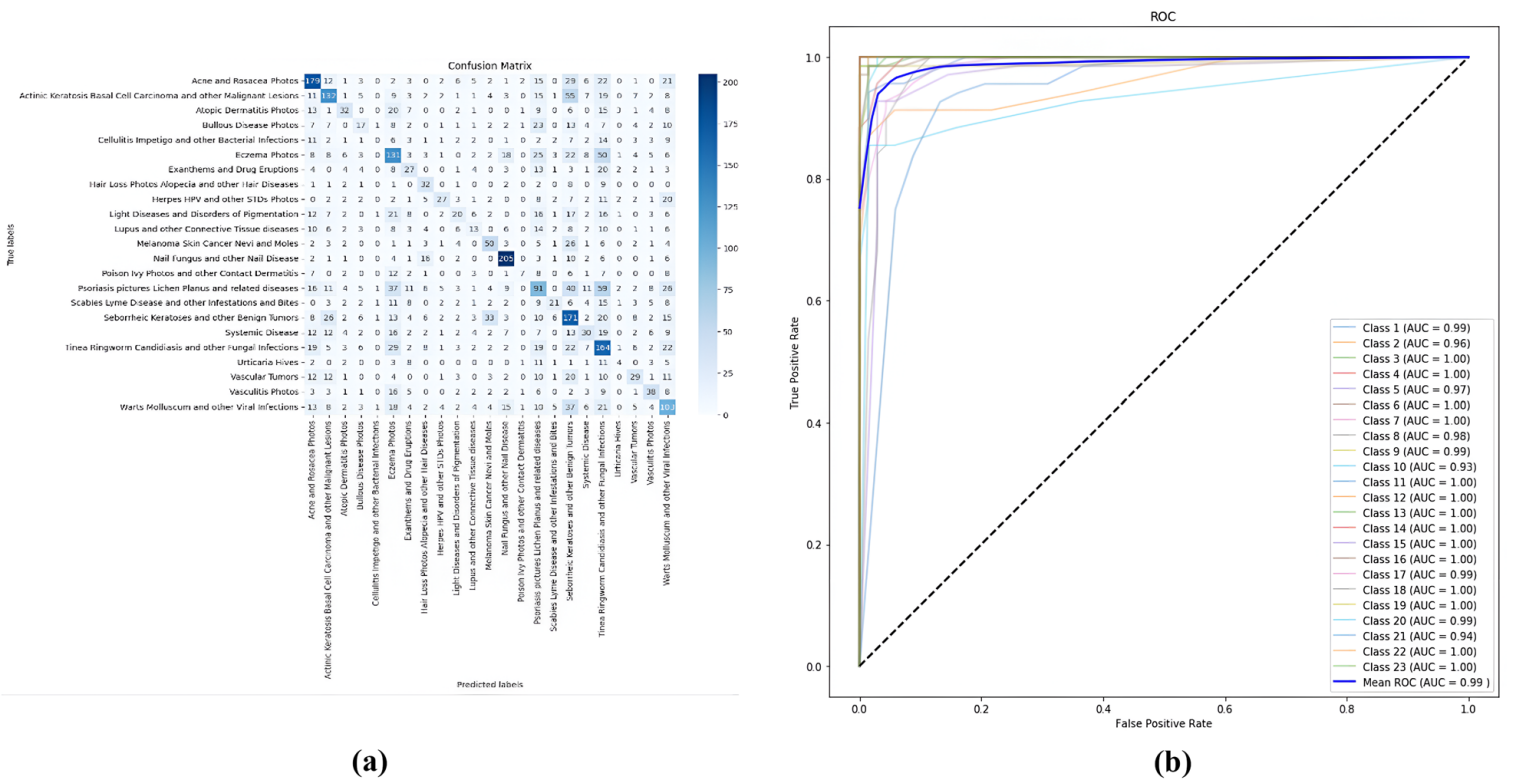
**(a)** Confusion matrix. **(b)** ROC curve.

The confusion matrix in Fig. 6a showcases minimal misclassifications in the 23 classes of diseases. Some common symptoms in Psoriasis and Eczema classes led to sporadic misclassifications.

The ROC curves demonstrated in Fig. 6b persistently display high AUC values. CHASHNIt exhibited values more than 0.95 for each category. This indicates its superior discriminative ability across various disease types. The comparative analysis of CHASHNIt with other models highlights its superiority in distinguishing between similar conditions.

All models including CHASHNIt, ResNet50, Swin Transformer and others were trained under identical conditions. Each experiment was repeated five times with different random seeds, and a mean ± 95 % confidence intervals was observed for accuracy, precision, recall, and F1-score. Paired t-tests indicate CHASHNIt’s accuracy is significantly higher than ResNet50 (p = 0.003) and Swin Transformer (p = 0.011). The IoU metric reported corresponds to Class Activation Map (CAM) overlap with dermatologist-annotated lesion masks, thereby justifying its use in a classification context.

### Comparative analysis

As it can be seen through Table 1, CHASHNIt outperforms the rest with 97.8% accuracy which is 7.6% points above the Swin Transformer and 8.2% above the ResNet101 highlighting better generalization and robustness in classification. While Swin Transformer with 90.2% accuracy demonstrates strong performance due to its multi-scale feature extraction capabilities, it still fails to match the overall effectiveness of CHASHNIt. ResNet101 and InceptionResNetV2, despite benefitting from residual connections and inception modules, achieve accuracy of 89.6% and 89.3%, respectively, indicating that their respective architectures are less optimized when independently used for dermatological image classification as compared to the hybrid fusion approach of CHASHNIt.

In terms of precision, CHASHNIt attains 98.1%, significantly outperforming Swin Transformer with 90.0% and ResNet101 with 88.3% precision scores. It indicates CHASHNIt’s worth in reducing false positives, which is a necessity in medical application where misclassification can induce unnecessary misclassifications or even wrong treatments. Furthermore, the recall score of 97.5% emphasizes the strength of CHASHNIt in classifying diseased cases when compared to other models such as Swin Transformer which is at 89.5% and ResNet101 which is at 89.7%, which tend to produce higher false negatives. The 97.6% F1-score, a balanced metric of precision and recall, emphasizes the high predictive reliability of the model, especially when compared to rival architectures that fail to achieve a 90% F1-score, with the Swin Transformer registering 89.8% and ResNet101 dropping to 87.4%. CHASHNIt records an IoU score of 92.3%, a huge leap from the Swin Transformer at 84.8% and ResNet101 at 86.0%, thereby registering a higher level of localization and segmentation accuracy in the classification of different types of skin diseases. MobileNetV3, EfficientNetB7, and DenseNet201 all record moderate performance, with different accuracies ranging from 87.5% to 88.7%. Conversely, ConvNeXt ranks at the lower end, with a lower accuracy of 86.4%, precision rate of 85.3%, and IoU score of 82.0%, thereby supporting its relatively lower performance in dermatological classification tasks.

**Fig. 7:**
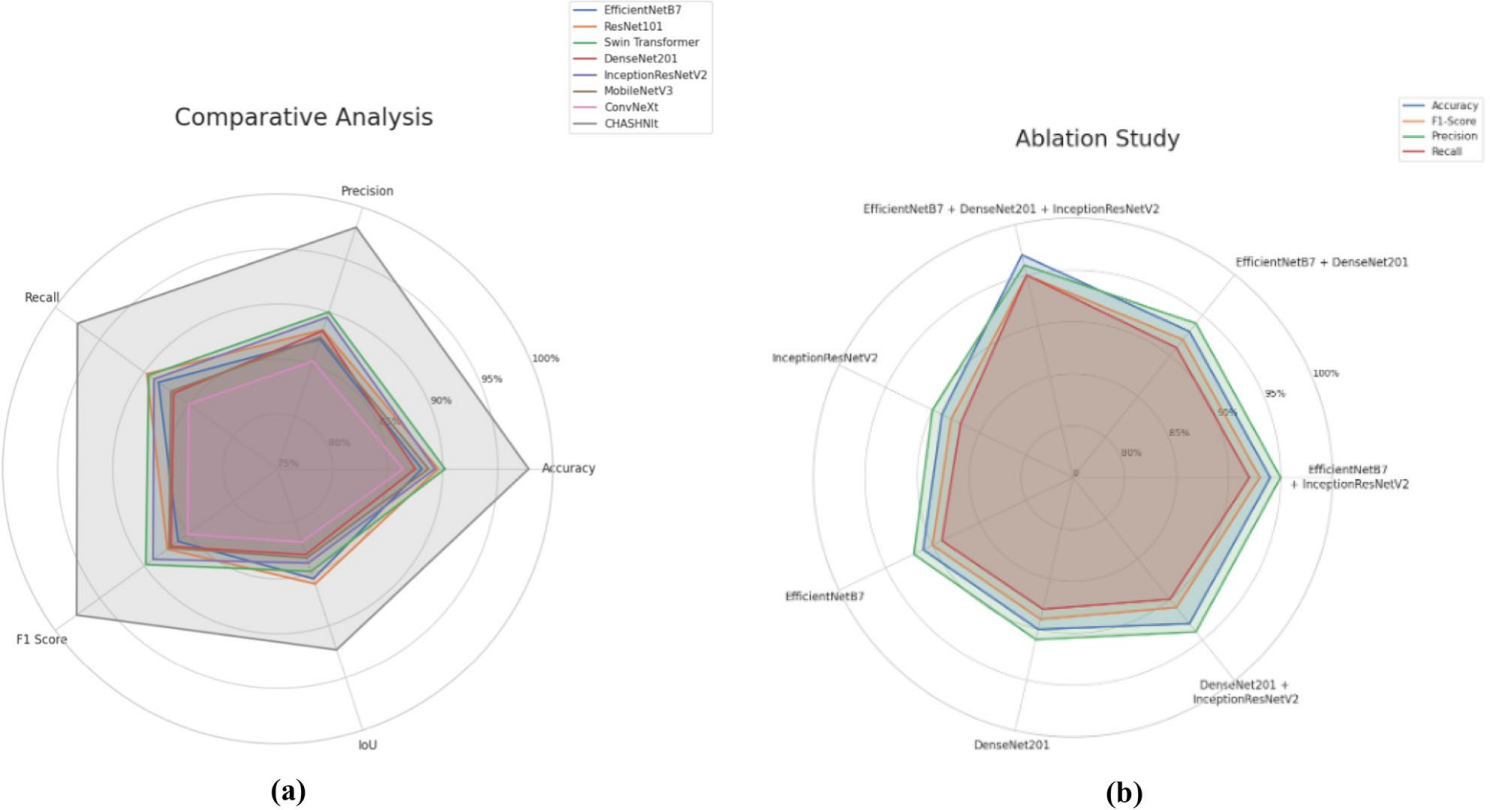
**(a)** Radial graph. **(b)** Ablation study.

The radial graph represented in Fig. 7a also shows how much CHASHNIt has stood its ground on all the parameters of performance. The shape on the graph that the model produces is well expanded and shows that the accuracy, precision, recall, F1-score, and IoU are all very strong with no significant drop in any area. The radial expansion of CHASHNIt directly correlates with its hybrid architecture’s ability to integrate diverse feature extraction methods, ensuring strong model consistency across all performance dimensions.

The ablation study as shown in Fig. 7b is conducted carefully to test the performance of all modules within the architecture of CHASHNIt, thereby further solidifying the superiority of its hybrid design. EfficientNetB7, which is famous for its compound scaling efficiency and depth-width-resolution trade-off optimization, achieves 88.1% accuracy, which lags 9.7 percentage points behind that of CHASHNIt. DenseNet201, with dense connectivity and gradient boosting, achieves 87.5% accuracy, denoting improvement in the reuse of features, although not in overall classification refinement. InceptionResNetV2, with the addition of inception modules and residual connections, performs marginally better with 89.3% accuracy; however, it still lags behind CHASHNIt by approximately 8.5 percentage points. This establishes the fact that none of these models are able to perform the same on their own as their hybrid counterpart.

In addition to measuring the performance of individual models, ablation study further clarifies notable improvements through feature fusion.

Overall, CHASHNIt establishes a new benchmark in artificial intelligence for dermatology, significantly outperforming all existing architectures. The comparison verifies its high accuracy, stability, and explainability, demonstrating its potential as a clinically effective deep-learning model. The radial graph visualization emphasizes CHASHNIt’s consistent superiority, while the ablation study emphasizes the importance of its hybrid architecture.

### XAI heatmap analysis

Two algorithmic frameworks namely LIME and SHAP were deployed to explain the decision making process of the models by generating heatmaps. The images start with the left section showing the original image, second section displaying the lime heatmap and third section with SHAP heatmap and desaturated version of the image without any heatmap overlay.

**Fig. 8:**
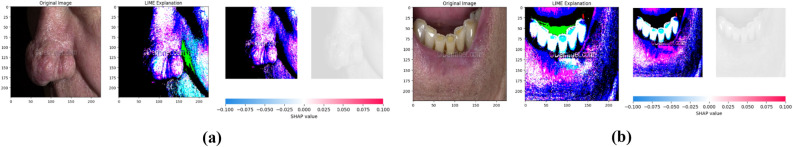
**(a)** Acne and rosacea. **(b)** Actinic keratosis basal cell carcinoma and other malignant lesions.

#### Acne and rosacea

The irregularities on the nasal tip and surrounding areas in the original image of Fig. 8a indicates the presence of acne or rosacea. The LIME heatmap highlights the regions towards left and right side of the nasal tip with purple tone indicating a significant contribution towards the diagnosis of disease. SHAP highlighted the affected regions near the tip and edges with purple signifying the contribution of the region in diagnosis.

#### Actinic keratosis basal cell carcinoma and other malignant lesions

The original image in Fig. 8b indicates the presence of Lesions int the labial mucosa and surrounding areas. LIME highlights the most affected areas in the left and right regions of lower vermilion in purple conveying utmost significance. SHAP highlights the affected regions of lower vermilion as well as the unaffected regions surrounding the middle of vermilion, lingual septum and labium inferius.

**Fig. 9:**
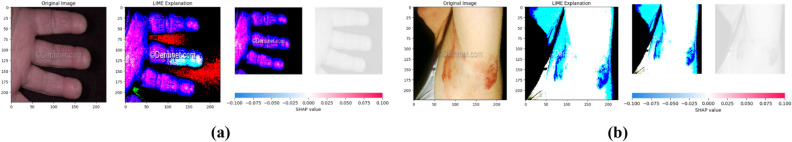
**(a)** Atopic dermatits. **(b)** Bullous disease.

#### Atopic dermatitis

The wrinkles on the skin in original image as demonstrated in Fig. 9a indicate the presence of Atopic Dermatitis on the fingers and surrounding areas. The LIME heatmap highlights the affected areas on two of the fingers in purple and the third finger in blue. Purple colour indicates the utmost significance of the region in diagnosis. Presence of red and green colour between the fingers indicates noise. SHAP highlights the inflamed and wrinkled areas along with some portion of unaffected areas near the palm.

**Fig. 10:**
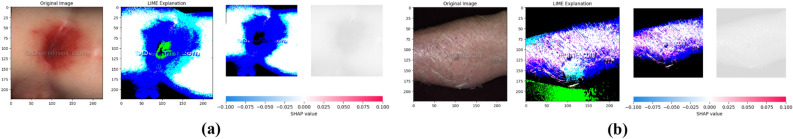
**(a)** Cellulitis impetigo and other bacterial infections. **(b)** Eczema.

#### Bullous disease

The red spots on the front and rear regions of axilla in the original image of Fig. 9b indicates the presence of Bullous disease. The LIME heatmap highlights the two majorly visible affected spots in blue and the other unaffected regions in white. SHAP accurately highlights all the affected regions in dark blue colour.

#### Cellulitis impetigo and other bacterial infections

Original image in Fig. 10a shows a bright red region surrounding the anal opening, indicating the effect of infection. LIME heatmap highlights the affected regions surrounding the opening in dark blue indicating utmost significance in detecting the disease. SHAP heatmap highlights the affected areas surrounding the centre as well as the unaffected region near the upper edge deprived of any swelling or redness. Both the frameworks also highlight some unaffected regions along the edges in blue.

#### Eczema

Wrinkles and scars in the original image in Fig. 10b indicate the presence of Eczema. The LIME heatmap in the second section of the image highlights some of the affected regions in purple and some in blue. SHAP heatmap demonstrates accurate colouration of affected regions in purple with the unaffected regions marked with white.

**Fig. 11:**
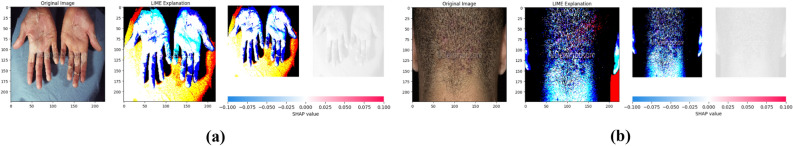
**(a)** Exanthems and drug eruption. **(b)** Hair loss alopecia and other hair diseases.

#### Exanthems and drug eruptions

The blisters and rashes in original image of Fig. 11a signify the effects of Exanthems and Drug Eruptions. LIME heatmap highlights the affected regions in the fingers in blue but does not highlight the affected regions near wrists and palm. SHAP highlights the affected regions in fingers as well as palms. The affected regions are accurately highlighted in dark blue colour indicating their significance in diagnosing the infection.

#### Hair loss alopecia and other hair diseases

The original image in Fig. 11b indicates hair loss and hair disease. The LIME heatmap highlights the affected regions with different colours. The SHAP heatmap majorly uses only three colours for highlighting the regions. This may cause ambiguity in identifying the affected regions.

**Fig. 12:**
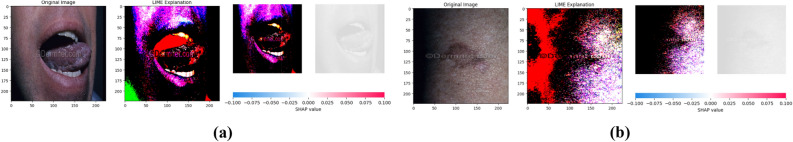
**(a)** Herpes and HPV, **(b)** light diseases.

#### Herpes and HPV

The presence of lesions near the regions surrounding lingual septum in the original image of Fig. 12a is a key symptom of Herpes or HPV. The LIME heatmap highlights the affected region in red colour beneath the tongue. SHAP framework accurately highlights the affected regions, mostly beneath the lingua. The most significant regions are highlighted in red and the most insignifcant are marked in black.

#### Light diseases (pigmentation disorderes)

Original image in Fig. 12b demonstrates a pigmentation disorder as a dark coloured irregular spot on the skin. The LIME heatmap highlights some of the portion of the image in dark colour. SHAP heatmap highlights only the right portion of the affected region. The black coloured area in the heatmap indicates the region marked as insignificant by the algorithm.

**Fig. 13:**
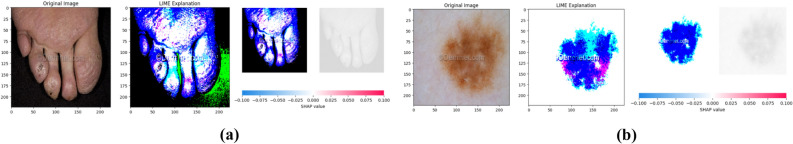
**(a)** Lupus and other connective tissue diseases. **(b)** Melanoma.

#### Lupus and other connective tissue diseases

The rashes demonstrated in the original image in Fig. 13a indicate the presence of connective tissue disease. The LIME heatmap highlights the rash on the second toe in purple and the other rash on the third toe in blue. SHAP framework highlights the both the rash accurately in purple.

#### Melanoma

The red coloured inflamed spot on the skin as shown in the original image in Fig. 13b is a visible symptom of Melanoma which plays a key role in the diagnosis of the disease. The LIME framework accurately highlights the affected areas in different colours based on the degree of redness. The SHAP heatmap highlights the entire affected area in same colour.

**Fig. 14:**
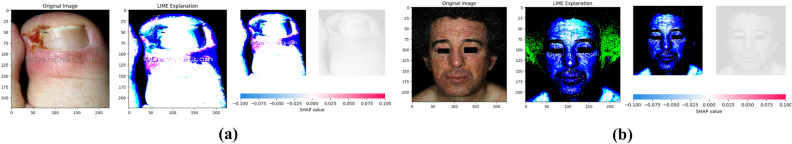
**(a)** Nail fungus and other nail diseases. **(b)** Poison ivy and other contact dermatitis.

#### Nail fungus and other nail diseases

The bright red coloured spot on the lateral nail fold as shown in the image in first section of Fig. 14a is a key symptom of nail fungus. LIME heatmap highlights the affected areas in dark blue colour. The SHAP framework highlights the whole affected region in a smooth form.

#### Poison ivy and other contact dermatitis

The hydropic blisters and rashes on the forehead and cheeks as shown in Fig. 14b indicate the presence of poison ivy dermatitis. The LIME heatmap highlights the affected regions around the buccal and either sides of the forehead in dark blue colour. The SHAP framework accurately highlights the presence of rashes and blisters in dark blue colour.

**Fig. 15:**
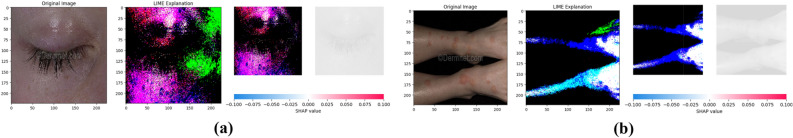
**(a)** Psoriasis and lichen planus. **(b)** Scabies and other infestations.

#### Psoriasis and lichen planus

The scaly areas on the palpebra in the original image in Fig. 15a are key symptoms of psoriasis and lichen planus. The LIME heatmap highlights the lower regions of the upper palpebra distinctly. The emphasis on these regions make them highly significant for diagnosis of the disease. The SHAP heatmap highlights the affected areas accurately without any presence of noise in the image. The algorithm significantly brings out the presence of lesions on the skin.

#### Scabies and other infestations

Light red coloured inflammatory spots spread across the legs in the original image of Fig. 15b indicate the presence of infestations. The LIME heatmap highlights some of the regions of the left leg in dark blue. It highlights the affected regions on the right leg in light blue colour marking them as medically insignificant as compared to the other affected areas. The SHAP heatmap indiscriminately highlights the affected regions of both the legs in dark blue colour. The algorithm considers all of the affected regions at same level of significance.

**Fig. 16:**
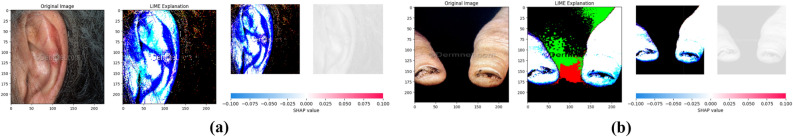
**(a)** Seborrheic keratoses and other benign tumors. **(b)** Systemic diseases.

#### Seborrheic keratoses and other benign tumors

The rotund skin outgrowth near the concha in the original image in Fig. 16a is a key symptom of seborrheic keratoses. The LIME heatmap highlights the affected spot with white and light blue colours. The framework identifies the circular highlighted region as a significant factor in identifying the disease. The SHAP framework highlights the affected region near the concha accurately and distinctively. The algorithm marked the other unaffected regions with different colour, reducing the chances of misdiagnosis.

#### Systemic diseases

The black pigmentation on the lateral nail fold and distal edge int the first image of Fig. 16b is a key symptom of systemic disease. The LIME heatmap highlights the affected regions near the folds and nail body in dark blue colour. The SHAP framework highlights the affected areas accurately in dark colours. The regions comprising of lateral nail folds, nail body and distal edge are considered significant in identifying the disease.

**Fig. 17:**
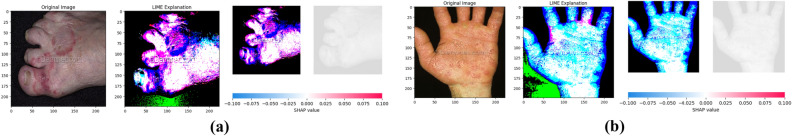
**(a)** Tinea ringworm and other fungal infections. **(b)** Urticaria hives.

#### Tinea ringworm and other fungal infections

The original image in Fig. 17a demonstrates the presence of red lesions following a ring shaped pattern, this is a key symptom of fungal infections like ringworm. The LIME heatmap highlights the affected regions in dark purple shade. The SHAP algorithm accurately highlights the affected areas on the toes and regions surrounding deep peroneal in purple colour.

#### Urticaria hives

The presence of red coloured welts in the first image of Fig. 17b plays an important role in diagnosing urticaria hives. The LIME heatmap highlights the affected regions in a disintegrated manner with light blue coloured spots across the area. The SHAP framework accurately highlights affected region with light blue coloured spots on the palm.

**Fig. 18:**
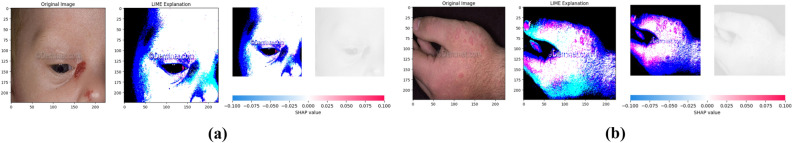
**(a)** Vascular tumors. **(b)** Vasculitis.

#### Vascular tumors

The red lesion covering the region around superior lacrimal in the original image in Fig. 18a indicates the presence of vascular tumor in the region. The LIME heatmap highlights the affected regions in dark blue colour. According to the heatmap, the framework identifies the regions near the superior lacrimal and nasal tip as significant for diagnosis of the tumor. The insignificant regions are highlighted in light blue and white colour. SHAP algorithm highlights the affected region near the lacrimal and nasal tip with dark blue colour in a continuous pattern.

#### Vasculitis

Small light red coloured spots near the radial in the first image of Fig. 18b play an important role in diagnosing vasculitis. The LIME framework highlights the affected regions in purple colour. The regions near thumb are highlighted as significant regions for the identification of the disease. LIME tends to ignore the affected region lying between the knuckles and the radial by highlighting them in light blue colour. SHAP’s heatmap accurately highlights all the affected regions in purple. The framework highlights the circular spots efficiently, considering all of the affected spots at the same level of significance.

#### Warts molluscum and other viral infections

**Fig. 19:**
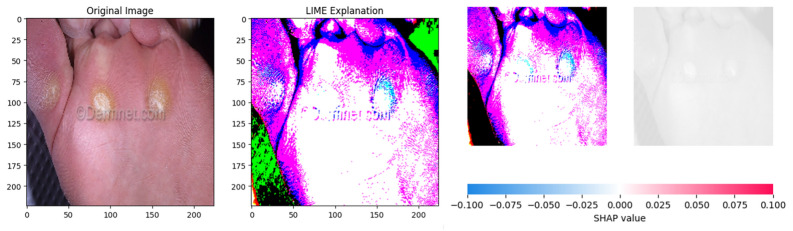
LIME and SHAP heatmaps for warts molluscum and other viral infections.

The original image in Fig. 19 demonstrates the presence of bristles on the skin which is a key symptom of warts and other viral infections. The LIME heatmap highlights the two of the affected spots in light blue colour. The algorithm misses the affected region in lower medial forefoot and highlights the spot in white marking it as insignificant. The green patches in the either sides of the image indicate the presence of noise in the image. The SHAP heatmap identifies and highlights all of the affected regions in light blue colour. Highlighted regions comprise of medial and lateral forefoot. The insignificant areas are highlighted in purple and white colours.

Analytical comparison of the two frameworks, SHAP and LIME indicates SHAP’s superior performance as compared to LIME. SHAP demonstrated more accurate and clinically significant outcomes, while LIME was more sensitive to noise which often affected the interpretability. The explainability outputs of CHASHNIt using both LIME and SHAP was evaluated. Quantitative overlap with dermatologist-annotated masks is reported in Section 3. SHAP achieved a mean IoU of 0.68 ± 0.04, whereas LIME scored 0.62 ± 0.05 (p< 0.01, Wilcoxon signed-rank test). Clinician relevance scores (1–5 scale) averaged 4.1 ± 0.3 for SHAP and 3.7 ± 0.4 for LIME (n = 200; p = 0.02), indicating that dermatologists found SHAP slightly more informative.

This analysis highlights the role of explainability utilities in ensuring reliability and trust in models. CHASHNIt outperforms all existing models in skin disease classification, yielding unparalleled outcomes in accuracy and XAI integration. The analysis highlights its practical utility, clinical significance, and potential in advancing dermatological diagnosis. Future work could focus on optimizing the model for diverse datasets to enhance its generalizability and clinical implementation.

## Conclusion

This work introduces CHASHNIt, a Combined Hybrid Architecture for Scalable High-performance in Neural Iterations, as a novel deep learning model for skin disease classification, tackling important issues like data imbalance, interpretability, and classification accuracy. The combination of EfficientNetB7, DenseNet201, and InceptionResNetV2 leverages the unique strengths of each architecture using feature extraction, achieving significant classification accuracy enhancement. GAN-based augmentation makes it possible to alleviate class imbalance by generating high-quality synthetic images, which guarantees representative and balanced coverage of all 23 classes of diseases. The addition of Explainable AI techniques increases the model’s usability in a clinical context by SHAP and LIME, which boost decision-making transparency. The suggested framework exhibits outstanding performance with accuracy at 97.8%, precision rate at 98.1%, recall rate at 97.5%, and Intersection over Union or IoU of 92.3%, hence outperforming top models such as Swin Transformer, ResNet101, MobileNetV3, and others. Exhaustive ablation study supports the synergistic gain of the hybrid approach, substantiating the enhanced performance of the model across a range of performance metrics. The efficacy of GAN augmentation and sophisticated preprocessing is also established via comparison, further supporting the reliability of the strategy. In spite of the strengths noted, some obvious limitations are evident. The use of a single information source, in this instance, DermNet, may limit generalizability of findings to other populations and to other imaging diseases. Furthermore, the significant computational demands of the hybrid model also raise questions about practical usability in resource-limited real-world settings. These are areas that underscore the necessity to continue to advance practical usability.

Future research will focus on improving computational efficiency by optimizing network architecture and simplifying the model without compromising performance, as well as conducting multi-center clinical trials to assess CHASHNIt’s generalizability across diverse geographic regions and imaging devices. Additionally, deploying CHASHNIt as a cloud-based diagnostic tool is proposed to support small clinics lacking dedicated GPU infrastructure. Leveraging recent advances in SHAP-guided, energy-efficient cloud scheduling^31^, this approach aims to enable real-time, interpretable predictions with minimal resource overhead.

With its established accuracy, interpretability, and class coverage, CHASHNIt is a candidate as a clinical decision-support instrument for dermatology. With its SHAP and LIME usage, it facilitates explainable predictions which is a critical element of clinical trust and its performance on 23 classes renders it deployable in real-world environments, particularly for assist triage or second-opinion use cases. While broader multi-center validation and deployment testing are future goals, CHASHNIt is a technically valid and clinically relevant beginning for explainable AI use in dermatology.

## Data Availability

The datasets used and analysed during the current study are publicly available from the DermNet repository at http://www.dermnet.com/dermatology-pictures-skin-disease-pictures.
